# A Decision Risk Assessment and Alleviation Framework under Data Quality Challenges in Manufacturing

**DOI:** 10.3390/s24206586

**Published:** 2024-10-12

**Authors:** Tangxiao Yuan, Kondo Hloindo Adjallah, Alexandre Sava, Huifen Wang, Linyan Liu

**Affiliations:** 1 School of Mechanical Engineering, Nanjing University of Science and Technology, Nanjing 210094, China; liulinyan@njust.edu.cn; 2 LCOMS, University of Lorraine, 57078 Metz, France; kondo.adjallah@univ-lorraine.fr (K.H.A.); alexandre.sava@univ-lorraine.fr (A.S.)

**Keywords:** risk, framework, decision-making, data quality issues, sensor

## Abstract

The ability and rapid access to execution data and information in manufacturing workshops have been greatly improved with the wide spread of the Internet of Things and artificial intelligence technologies, enabling real-time unmanned integrated control of facilities and production. However, the widespread issue of data quality in the field raises concerns among users about the robustness of automatic decision-making models before their application. This paper addresses three main challenges relative to field data quality issues during automated real-time decision-making: parameter identification under measurement uncertainty, sensor accuracy selection, and sensor fault-tolerant control. To address these problems, this paper proposes a risk assessment framework in the case of continuous production workshops. The framework aims to determine a method for systematically assessing data quality issues in specific scenarios. It specifies the preparation requirements, as well as assumptions such as the preparation of datasets on typical working conditions, and the risk assessment model. Within the framework, the data quality issues in real-time decision-making are transformed into data deviation problems. By employing the Monte Carlo simulation method to measure the impact of these issues on the decision risk, a direct link between sensor quality and risks is established. This framework defines specific steps to address the three challenges. A case study in the steel industry confirms the effectiveness of the framework. This proposed method offers a new approach to assessing safety and reducing the risk of real-time unmanned automatic decision-making in industrial settings.

## 1. Introduction

**Figure 1:**
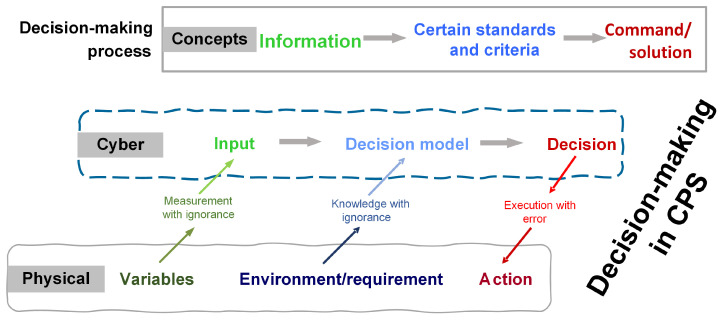
Decision-making process with CPS.

The development of cyber–physical systems (CPSs) and the Internet of Things (IoT) have enabled real-time measuring and analyses of more and more parameter data in the manufacturing industry at an acceptable cost, stimulating the development of increasingly advanced autonomous decision-making processes [1,2]. Autonomous manufacturing systems rely on advanced, big, data-driven, computer-based decision-making methods to achieve high production performance, especially in poor working environments. However, as shown in Figure 1, when detection, decision models, and execution are biased, the entire control process will face unknown responses from physical entities.

In the context of green energy-saving requirements, some steel companies seek more energy-efficient control strategies for the cooling process. While attempting to use computer control for cooling towers, the authors encountered issues related to earning users’ trust during the development of adaptive control software for cooling tower management in steel companies. These issues can be categorized into the following aspects.

The first aspect is whether the decision model can adapt to the current, permanently existing random measurement deviations (measurement uncertainties) on-site. Under the conditions of the current measurement accuracy level of the system, how can the potential losses that may result from using this model be evaluated? The second issue concerns sensor selection and maintenance reminding. To allocate maintenance resources effectively, it is essential to identify which sensors have a high sensitivity to measurement accuracy degradation and further determine the maximum acceptable accuracy degradation for these specific sensors. The third aspect is whether the losses remain controllable when on-site sensors experience faults or information missing in continuous processing.

The commonality among these three aspects lies in the potential loss issues caused by data quality problems in the context of automated continuous control scenarios. We will now discuss these issues from the perspectives of data quality, potential losses, and fault-tolerant control.

### 1.1. Data Quality Issues

Zaveri et al. [3] identified two dimensions in data quality: the first is the inherent quality of data, and the other is the quality of the data context. A more widely accepted definition of data quality involves assessing whether a piece of data meets users’ information requirements in a specific application [4]. Yuan et al. [5] reviewed 24 data quality characteristics from different aspects. Table 1 lists the classification of data quality characteristics in industrial decision-making scenarios in terms of inherent and system-dependent types.

The data usage scenario in this article pertains to real-time decision-making. The data considered in this article are sensor data. When evaluating the impact of data quality issues on real-time decision-making, the data quality problem can be regarded as a measurement error, defined as the difference between measured values and reference quantity values [6]. Sensor data quality issues come from two main sources: permanent measurement uncertainty and occasional sensor failures.

### 1.2. Persistent and Non-Negligible Measurement Uncertainty

When using sensors for measurement, uncertainty is always present. The *Guide to the Expression of Uncertainty in Measurement* (*GUM*) [7] defines uncertainty as “the degree of dispersion that can be reasonably attributed to the quantity value associated with a parameter related to the measurement result”.

In the steel industry, to precisely control equipment temperature and reduce scaling, a high-flow, low-temperature differential water cooling process is commonly used. This method controls the temperature variation between the supply and return water to within 5 °C. However, due to the cost considerations of sensor placement, commonly used temperature sensors such as thermocouples have a relatively large measurement uncertainty of about 1.5 °C. Some scholars refer to the potential impact of this measurement uncertainty as the “elephant in the room” [8]. In this case, measurement uncertainty cannot be ignored.

### 1.3. The Tremendous Destructiveness of Sensor Failures in Continuous Decision-Making

In practical applications, sensors are prone to failures. Common sensor failure modes include failure, bias, drift, and accuracy degradation [9]. Automated equipment may continuously make decisions based on faulty sensor data, leading to a cumulative effect of incorrect decisions that could trigger broader systemic cascading effects and potentially cause catastrophic failures. For instance, on 29 October 2018, shortly after takeoff, Indonesian Lion Air Flight JT610 encountered a malfunction in its Angle of Attack (AOA) sensor, causing the anti-stall system to misidentify the aircraft’s normal flight condition as a stall and making erroneous automatic adjustments, ultimately leading to the plane’s crash [10]. Therefore, in systems with continuous automated decision control and less human oversight, the consequences of sensor failures can be disastrous.

In the process of building automated decision-making models, robust optimization methods are widely applied to address uncertainties in system inputs [11]. Especially in risk-sensitive fields such as drones, autonomous driving, and nuclear power, decision control mechanisms that involve emergency shutdowns and automatic switches to a safe mode are employed to minimize the probability of potential accidents and reduce environmental impact [12]. However, in the continuous production lines of the steel industry, even if a sensor fails, it is typical not to halt the entire production line. Thus, sensor fault-tolerant control needs to consider decision-making with the production line continuing to operate after a sensor failure.

### 1.4. Risk: Potential Losses

In the design and application process of automatic control systems, users always attach great importance to the potential losses they might suffer. Therefore, in the decision-making process, we adopt risk as the key indicator. Different industries have different definitions and understandings of “risk”, with some fields, like aircraft, equating risk to the likelihood of disaster events occurring [13], while other fields, like environment, consider risk as the economic losses caused by disaster events [14]. In engineering practice, “accident” is often used to describe risk. Tools or methods such as failure mode and effects analysis (FMEA) [15] and risk matrix [16] are commonly adopted to evaluate and prioritize multiple possible accidents.

The risk mentioned in this article specifically refers to the losses caused by incorrect decisions during the control process. The measurement of risk is derived from the result of multiplying the possibility of an accident occurring by its potential damage estimate. For a continuous control decision-making environment, accumulating the risk after each choice or strategy implementation can depict the total potential risk throughout the decision-making process.

### 1.5. Necessity of Risk Assessment Framework

In a traditional manufacturing environment, once the production process is set, key operating parameters are fixed (or change fixedly over time), and the stability of the environment and input conditions is maintained as much as possible, which avoids risks associated with real-time variable control.

In contrast, future smart manufacturing environments require more flexibility, with real-time adjustments to product or environmental parameters becoming the norm to achieve energy savings or adapt to constantly changing control demands. In such scenarios, controlled parameters need to be frequently adjusted to respond to ongoing changes.

In the current decision-making risk assessment, the primary areas of research focus on investment [17,18], policy development [19,20], and project management [21,22]. However, there is a significant gap in research addressing the quality of decision input data within the field of risk assessment. Comprehensive studies that consider data quality, decision biases, and their impact on risk assessment are notably scarce. This underscores the urgent need for real-time evaluations of how data quality issues influence risks in the continuous decision-making process.

### 1.6. Conclusions

Motivated by the above, this article proposes a decision risk evaluation and alleviation framework. The main contributions are as follows:

1. Risk is defined as potential loss and indicates the decision-making model’s performance. A risk assessment and reduction framework is established at three levels: perception, decision, and execution, focusing on on-site data quality issues and associated risks.
2. The risk level is evaluated by Monte Carlo simulation considering the quality of on-site measurements and typical working conditions.
3. Based on this framework, three applications are introduced.

The rest of this paper is organized as follows. Section 2 presents the problem statement. Section 3 constructs a risk assessment and reducing framework related to data quality issues. Section 4 provides a case study to demonstrate the effectiveness of the proposed method. Finally, Section 5 presents the conclusion.

## 2. Problem Statement

**Figure 2:**
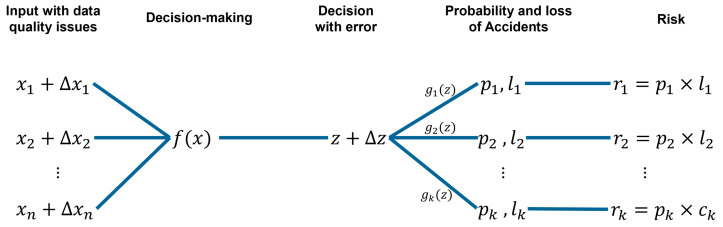
Decision-making and risk assessment scenarios under data quality influence.

The research work introduced in this paper aims to propose a framework for evaluating the decision risks induced by data quality problems in scenarios of automatic control of industrial production systems from models. Figure 2 illustrates the entire scenario, from raw data to decision risk assessment through decision-making.

where:

 *n* is the number of variables input data to the decision model;
 $x_{i}$ is the $i^{th}$ input variables of the, the decision model, i=1,⋯,n;
 $\Delta x_{i}$ is the difference between true value and measured values, i=1,⋯,n; it contains the data quality characteristics;
 *k* is the number of potential accidents identified caused by the faulty data in the decision-making model;
 *z* is the output of the decision model;
 Δz is the output bias of the decision model;
 $g_{j}\left(z\right)$ is the relation model between the decision z+Δz and the probability of the $j^{th}$ accident, j=1,…,k;
 $p_{j}$ is the probability of the $j^{th}$ accident, j=1,…,k;
 $l_{j}$ is the $j^{th}$ damage cost due to the $j^{th}$ accident, j=1,…,k;
 $r_{j}$ is the potential loss (risk) that the system faced by $j^{th}$ accident, j=1,…,k.

### 2.1. Assumption of Decision-Making Model

Let us assume that the decision model is defined prior to the assessment and that the input variables of the decision model are $x_{1},x_{2},\cdots x_{n}$. In this scenario, the decision model is described by the function *f* in Equation (1), where *z* is the output of the decision function.

(1) $$z=f\left(x_{1},x_{2},\cdots x_{n}\right)$$

where:

 $x_{1},x_{2},\cdots x_{n}$ are the input variables of the decision model;
 *z* is the output of the decision model.

Due to measurement inaccuracies in the input variables, there is inherent uncertainty $\Delta x_{i}$ associated with each input, which affects the decision model’s output variable by Δz:

(2) $$z^{\prime }=f\left(x_{1}+\Delta x_{1},x_{2}+\Delta x_{2},\ldots ,x_{n}+\Delta x_{n}\right)$$

where $z^{\prime }=z+\Delta z$.

### 2.2. Assumption of Data Quality Issues

Sensor data are considered in this work, where data quality problems can be regarded as the difference between the measured value and true value. So, let us assume that both the instrumental uncertainty and the measurement deviation due to degradation of the sensor’s accuracy are normally distributed. Based on the prediction results, starting from historical data under typical operating conditions, the distribution of differences between the assigned values and the actual values after imputing data lost due to sensor faults was evaluated. Another assumption is that no issue, such as data loss due to network transmission, could affect data quality.

### 2.3. Assumption of Risk Identification and Evaluation Model

Let us assume that the relation between risk and decision bias has been obtained through external experiments. We define the risk in this work as potential severe damage or loss resulting from an unforeseen and unwanted event. The risk can be calculated by multiplying the event probability by the estimated cost of the damage or loss, as proposed by Ni et al. [16], as expressed in Equation (3).

(3) Risk=(probability of hazard)×(estimated loss of damage)

Due to the difference Δy between the model’s output and the actual value, the probability function governing the event *i* occurrence can be expressed by Equation (4).

(4) $$p_{i}=g_{i}\left(z,\Delta z\right)$$

In a scenario with *k* potential independent events, the probability and damage or loss by each event *i* are $p_{i}$ and $l_{i}$, respectively. The total systemic damage balance including losses relating to all individual risks $r_{i}$, i=1,…,k, are then given by:

(5) $$R=\sum_{i=1}^{k}r_{i}=\sum_{i=1}^{k}p_{i}l_{i}=PL^{T}$$

where $P=\left[p_{1},p_{2},\ldots ,p_{k}\right]$ and $L=\left[l_{1},l_{2},\ldots ,l_{k}\right]$.

Considering the decision-making cycle, the decision risk over a time horizon *T* is aggregated from the risks relating to each decision-making time cycle $t_{i}$ for each risk *i*:

(6) $${\bar{R}}_{\mathrm{cycle}}=\frac{1}{k}\sum_{i=1}^{k}\left(\frac{T}{t_{i}}\times r_{i}\right)$$

where ${\bar{R}}_{\mathrm{cycle}}$ is the average risk over the meantime cycle on the time horizon *T*.

## 3. Risk Assessment and Alleviation Framework

**Figure 3:**
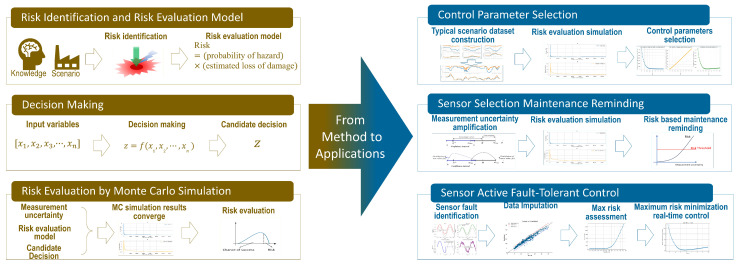
Risk assessment and alleviation framework.

Since risk is always linked to unpredictable random events, one can base its anticipation on predictions through simulations based on data collected on its influence parameters. Here, one is particularly interested in decision risk linked to data quality failures. Figure 3 shows the proposed risk assessment and alleviation framework. The brown section on the left describes the three main stages of assessment of decision risk: risk identification, decision-making, and decision-making risk assessment through Monte Carlo simulations. The blue part on the right shows the three applications of risk alleviation based on decision-making risk, including control parameter selection, sensor selection, sensor selection maintenance reminding, and sensor active fault-tolerant control.

### 3.1. Decision Risk Assessment by Monte Carlo Simulation

Algorithm 1 describes a structured approach to making decisions under uncertainty by using a decision model and then evaluating the related risks using Monte Carlo simulation. The steps involve generating decision outputs, assessing the risks iteratively, and finally relating the calculated average risk with the decision.
**Algorithm 1** Risk Assessment for Decision under Uncertain Information  1:**Step 1: Make a decision based on current information and decision model**  2:**Input:** Current Information $x_{1},x_{2},\ldots ,x_{n}$, Decision Model *f*  3:**Output:** One Decision *z*  4:**Action:**  5:   Decision ← Decision Model (Information): $z=f(x_{1},x_{2},\ldots ,x_{n})$  6:**Step 2: Perform Risk evaluation by Monte Carlo simulation**  7:**Input:** Decision, Risk Assessment Model (R), Data Quality Features  8:**Output:** Simulated Risk Averages for Each Decision  9:**Action:**10:   Initialize Risk_Results ←[]11:   Construct distribution and variance for data with quality issues (Decision, Data Quality Features)12:**while** simulation results have not converged **do**13:       Generate random data samples (Sample_Data) within the uncertainty range14:       Perform risk assessment:15:          Risk←R(Sample_Data, decision)16:          Add Risk to Risk_Results17:**end while**18:   Calculate the simulated risk average for this decision:19:      Risk_Average← Calculate_Average(Risk_Results)20:   Associate Risk_Average with this decision

### 3.2. Risk Alleviation Applications

The following subsection introduces three potential applications of risk alleviation regarding data quality issues.

#### 3.2.1. Control Parameter Selection under Persistent Measurement Uncertainty

##### Step 1: Typical Scenario Dataset Construction

Evaluation based on typical operating conditions is an important step in engineering, testing, and performance evaluation [23]. The core task of constructing a typical working condition dataset is to identify several representative conditions and calculate the proportion of each condition in the current scenario, ultimately generating a dataset that accurately reflects the working conditions of the scene. Existing research on the construction of typical working conditions is primarily focused on vehicle driving conditions in urban traffic. Common algorithms used for this purpose include K-Means [24], Density-Based Spatial Clustering of Applications with Noise (DBSCAN) [25], and Principal Components Analysis (PCA) [26], among others.

##### Step 2: Advice for Parameter Selection

In typical operating conditions, the data are a measured value with uncertainty collected by a sensor. The true value is unknowable, so we generate a random measurement uncertainty based on the measurement uncertainty σ in the sensor’s own parameters and load it on the measured value *x* to generate the true value $x^{*}$. The true value $x^{*}$ can be modeled as:

(7) $$x^{*}=x+\epsilon$$

According to *GUM* (*Guide to the Expression of Uncertainty in Measurement*), when the sensor’s expanded uncertainty factor k=2, the true value has a 95.4% probability of falling within the measured value plus or minus the 2 σ confidence interval. The uncertainty ϵ can be modeled as normally distributed with zero mean and standard deviation σ, i.e., $\epsilon ∼N(0,\sigma^{2})$. Thus, the 95.4% confidence interval for $x^{*}$, based on the measurement *x*, is given by:

(8) $$P(x-2\sigma \leq x^{*}\leq x+2\sigma )=0.954$$

We will input the measured value and generate the “true value” into the decision-making process, compare the decision biases, and then calculate the risk accordingly. In each decision-making simulation cycle, random data will be generated many times until the simulation results converge. Then, the statistical characteristics of the expected risk ${\bar{R}}_{cycle}$ can be obtained over a time cycle.

Then, we can assess the risk under the current measurement uncertainty. Depending on the measured uncertainty of the current sensor, the control parameters $z^{*}$ that minimize the expected risk ${\bar{R}}_{cycle}$ are selected.

(9) $$z^{*}=\arg\min_{z}{\bar{R}}_{cycle}\left(z\right)$$

#### 3.2.2. Sensor Selection and Maintenance Reminding

First, the uncertainty related to each sensor input is incrementally amplified. Subsequently, these amplified uncertainties are considered within a Monte Carlo simulation of risk, based on a typical operational dataset, allowing for the observation of risk variations for each sensor under different levels of uncertainty.

For sensor selection, this information allows for the optimization of sensor accuracy within a limited budget across the system. The objective functions are expressed in Equation (10).

(10) $$\left\{\begin{array}{cc} \min C_{\mathrm{cycle}} & =\min\sum_{i=1}^{n}c_{i},\\ \min{\bar{R}}_{cycle} & =\min{\bar{R}}_{cycle}\left(\epsilon_{1},\epsilon_{2},\ldots ,\epsilon_{i},\ldots ,\epsilon_{n}\right) \end{array}\right.$$

where $C_{\mathrm{cycle}}$ is the budget for all sensors, $c_{i}$ is the cost of the sensor *i*, and $\epsilon_{i}$ is the measurement uncertainty of sensor *i*.

For sensor maintenance, as the sensor’s lifespan advances, measurement uncertainty increases, enabling the assessment of related risks. When the average risk ${\bar{R}}_{cycle}$ exceeds a certain threshold ${\bar{R}}_{th}$, it is recommended to perform sensor maintenance. This approach ensures that maintenance is carried out when the risk is high, as inequality (11) expresses it.

(11) $$\;\mathrm{If}\;{\bar{R}}_{cycle}>{\bar{R}}_{th},\;\mathrm{call}\;\mathrm{for}\;\mathrm{maintenance}\;\mathrm{action}.$$

#### 3.2.3. Sensor Active Fault-Tolerant Control

This subsection proposes a method for handling the sensor failure control process, aiming to improve the system’s fault tolerance and risk management efficiency. First, a large amount of historical data is analyzed to statistically characterize the sensor’s life, fault category, and measurement deviation during failure. A statistical or machine learning model is implemented to impute the missing value of a specific sensor based on the coupling relationship between the sensors’ measurements, and the imputation uncertainty is obtained. Secondly, using the predicted sensor life and measurement deviation during the fault, the risk interval that may be faced in the future is determined through risk simulation. Finally, in the real-time fault detection and maximum risk minimization control strategy, different control parameters are adopted to determine the parameter value that can minimize the maximum risk. The Algorithm 2 works as follows.
**Algorithm 2** Sensor Active Fault-tolerant Control  1:**Step 1: Real-time Fault Detection and Virtual Measurements**  2:**Input:** Historical Data, Sensor Measurements, Coupling Relationships  3:**Output:** Assigned Sensor Values and Deviations  4:**Action:**  5:   Analyze historical data to generate models to predict sensor values with other sensor values (if possible)  6:   Detect faults in sensor measurements  7:   If sensor *k* fails at time *t*:  8:      Predict sensor value $x_{k}^{*}\left(t\right)+\Delta$ using operational sensors $x_{1}\left(t\right)$ to $x_{n}\left(t\right)$ excluding $x_{k}\left(t\right)$  9:   End If10:**Step 2: Fault Detection and Maximum Risk Minimization Control**11:**Input:** Sensor values, Assigned Sensor Values and Deviations, Control Parameters12:**Output:** Optimal Control Parameter $p^{*}$13:**Action:**14:   If a fault is detected15:      Uniformly sample forecast deviations $E_{i}∼U\left(E_{min},E_{max}\right)$16:   For each sampled deviation $E_{i}$:17:         Perform Risk Monte Carlo simulation with the current control parameter *p*18:         Calculate risk $R(E_{i},p)$19:         Select the deviation $E_{r-max}$ with the maximum risk20:   End For21:   Reintroduce $E_{r-max}$ into the simulation with different control parameters $p_{j}$22:   For each control parameter $p_{j}$:23:      Calculate $R^{\prime }\left(E_{r-max},p_{j}\right)$24:      Determine the optimal control parameter $p^{*}$25:   End For

Assume that $x_{i}\left(t\right)$ represents the measurement from sensor *i* at time *t*. If sensor *k* fails at time *t*, its value needs to be credited based on the readings of other operational sensors.

(12) $$x_{k}^{*}\left(t\right)=g\left(x_{1}\left(t\right),x_{2}\left(t\right),\ldots ,x_{k-1}\left(t\right),x_{k+1}\left(t\right),\ldots ,x_{n}\left(t\right)\right)\pm \Delta$$

where $x_{k}^{*}\left(t\right)$ is the assigned value for sensor *k*, Δ represents imputation uncertainty, and *g* is the imputation function, which might rely upon techniques such as weighted averages, linear regression, data reconciliation, or machine-learning methods to estimate $x_{k}\left(t\right)$ based on the readings from other sensors $x_{1}\left(t\right)$ to $x_{n}\left(t\right)$ excluding $x_{k}\left(t\right)$.

##### Step 2: Real-Time Fault Detection and Maximum Risk Minimization Control

When a sensor fault detection function is present in the scenario, the sensor’s measured value becomes unreliable once the fault is detected. At this point, the data estimation in Step 1 is used to obtain the sensor’s measurement information or the sensor’s measurement deviation is obtained from the sensor fault statistics, as shown in Equation (12).

Uniform sampling within the range of forecast deviations ±Δ is conducted, and the sampled deviations are introduced into the Monte Carlo simulation (with the current control parameters) to assess the risks posed by different forecast deviations to the system.

(13) $$E_{i}∼U\left(E_{\min},E_{\max}\right),\;i=1,2,\ldots ,N$$

(14) $$R_{i}=R\left(E_{i},P\right),\;i=1,2,\ldots ,N$$

(15) $$E_{\max}=\arg\max_{E_{i}}R\left(E_{i},P\right)$$

The forecast deviation $E_{\max}$ with the highest risk is selected and reintroduced into the risk Monte Carlo simulation using different control coefficients.

(16) $$R^{\prime }\left(E_{\max},P_{j}^{\prime }\right),\;j=1,2,\ldots ,M$$

This process ultimately determines the optimal control parameters $p^{*}$ as shown in Equation (17).

(17) $$z^{*}=\arg\min_{z}max\left(R_{total}\left(\pm \Delta \right)\left(z\right)\right)$$

## 4. Case Study from Steel Industry

### 4.1. Brief Introduction

**Figure 4:**
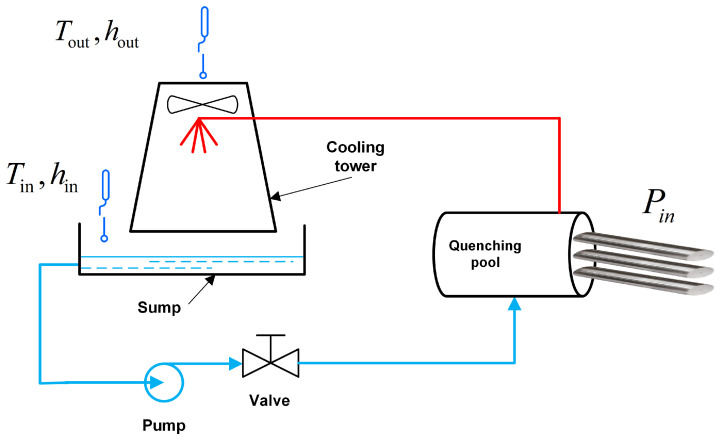
Illustration of the cooling process.

Let us illustrate this approach with a simplified example. Consider a steel plant where cooling systems utilizing heat exchange are essential for transferring heat from the continuous quenching station to the surrounding environment. The cooling system consists of a cooling tower, quenching pool, water pump, valves, and pipes. A schematic diagram of the operation of the system is shown in Figure 4. Currently, manufacturers are maximizing cooling tower ventilation, resulting in a great deal of wasted energy. The dynamic adjustment of cooling capacity optimizes energy consumption, but engineers are concerned about the risks associated with poor data quality in the field. Therefore, how to balance the demand for energy conservation and potential risks has become an important decision-making problem faced by the plant. The difficulty in the dynamic adjustment of cooling power lies in the fact that the temperature and humidity changes in the environment affect the cooling power. For example, when the humidity of the air inlet of the cooling tower is very high, the efficiency of water–gas heat exchange will be greatly reduced.

In practice, the calculation of the cooling rate of the cooling tower is very complex, and the flow rate of cooling water needs to be considered on the one hand, and the amount of ventilation in the pipe in the cooling tower needs to be considered on the other hand. So, let us simplify the control logic shown in Figure 4 to assume that the heat $P_{in}$, brought by hot steel, to be cooled is known in advance, and the air volume of the cooling tower adjusts the heat taken away by the cooling loop. This balances the heat brought by the hot steel and the heat taken away by the cooling tower.

(18) $$P_{in}=k_{r}Q$$

where $k_{r}$ is the redundancy of air volume and *Q* is the cooling power of the cool tower at a specific working condition.

The cooling power of the cooling tower *Q* is determined by calculating the difference between the enthalpy of the inlet air $E_{\mathrm{in}}$ and the enthalpy of the outlet air $E_{\mathrm{out}}$, and then multiplying this by the airflow rate passing through the tower *v*, as shown in Formula (19).

(19) $$Q=v\times (E_{\mathrm{out}}-E_{\mathrm{in}})$$

(20) $$E_{\mathrm{in}}=f_{\mathrm{enthalpy}}\left(T_{\mathrm{in}},h_{\mathrm{in}}\right)$$

By calculating the temperature and humidity of the inlet and outlet air by Formula (20), the enthalpy of the inlet air $E_{\mathrm{in}}$ and the enthalpy of the outlet air $E_{\mathrm{out}}$ can be obtained. See the Appendix A for a detailed calculation of the enthalpy difference.

(21) $$P_{\mathrm{in}}=k_{r}\times v\times \left(E_{\mathrm{out}}-E_{\mathrm{in}}\right)$$

From Formula (21), it can be seen that when there is a measurement deviation in the temperature and humidity sensors of the inlet and outlet $T_{\mathrm{in}},h_{\mathrm{in}},T_{\mathrm{out}},h_{\mathrm{out}}$, in order to ensure that the cooling tower provides sufficient cooling power in the system, the redundant $k_{r}$ of the airflow needs to be adjusted appropriately.

Data Collection

**Figure 5:**
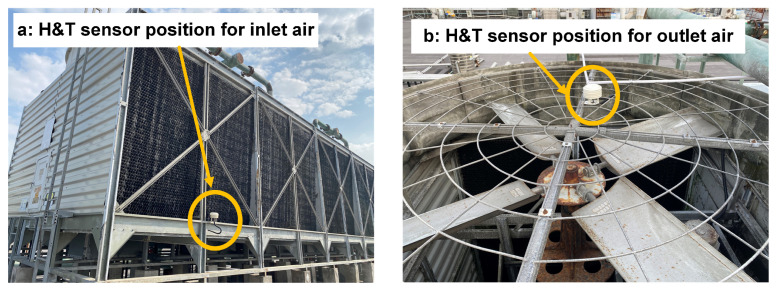
The position of the sensors on the cooling tower.

The values of $T_{\mathrm{in}}$, $h_{\mathrm{in}}$, $T_{\mathrm{out}}$, and $h_{\mathrm{out}}$ are collected by sensors. The T&H (temperature and humidity) sensor is an RS-BYH-M meteorological multi-element shutter sensor from Jiandarenke. The temperature measurement uncertainty is 0.5 K, and the relative humidity measurement uncertainty is 3% RH (RH∈[0,1]). The position of sensors is shown in Figure 5.

Risk Identification and Calculation

Let us define the cost unit by CU. The risk brought by the data quality allowed by the system is 1000 CU. Many risks exist in the considered system, caused by mechanical failure, imprecise control, or data quality issues. Additionally, two risks related to data quality issues and control are considered only in this case study for illustration. $r_{d}$ is the first risk due to uncertain control, which can result in a defective product. Steel quality is sensitive to temperature changes, which affect steel metallography and product quality. $r_{\mathrm{ew}}$ is the second risk, leading to wasted cooling energy. $r_{\mathrm{all}}$ is the sum of these two risks. The calculation process for each risk is detailed in the Appendix B.

### 4.2. Simulation

#### 4.2.1. Simulation for Risk and Fault-Tolerance Control

##### Step 1: Typical Scenario Dataset Construction

**Figure 6:**
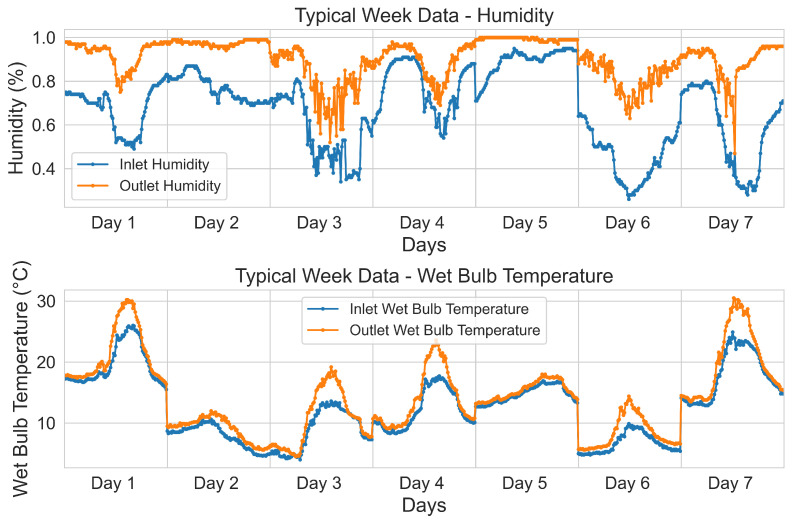
Typical week working condition.

The operating status of the cooling tower is mainly affected by air temperature and humidity. Assuming that the equipment is inspected once a week, we generated typical operating data samples based on the data within a week considered as the cycle. Therefore, in this study, we conducted a feature engineering analysis on the relationship between air temperature and humidity and machine operation. By analyzing the data from March and April, we used autoencoders to extract the features of the operating data adaptively. Based on these features, we used a Gaussian Mixture to classify the daily working conditions, and the classification results are plotted in Figure 6.

##### Step 2: Advice for Parameter Selection

**Figure 7:**
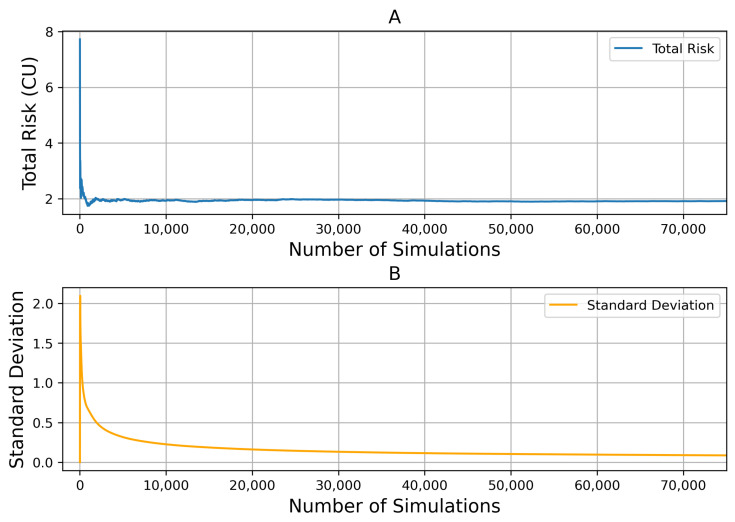
Monte Carlo simulation times. Subfigure (**A**) shows the convergence of total risk with Increasing Simulations. Subfigure (**B**) shows the standard deviation of total risk decrease with the number of Simulations.

The sensor’s parameters show that the temperature measurement uncertainty is 0.5K, and the relative humidity measurement uncertainty is 3%. According to the plan in Step 2, Section 3.2.1, control parameters are brought in randomly, and the amount of data generated is gradually increased to bring into the typical case dataset until the risk results converge and the number of Monte Carlo simulations is obtained. As Figure 7 shows, the simulation results tend to stabilize when the simulation times reach 20,000. Based on the number of current Monte Carlo simulations, different control parameters are brought into the simulation process to obtain the expected cumulative risk of different control parameters under typical working conditions.

**Figure 8:**
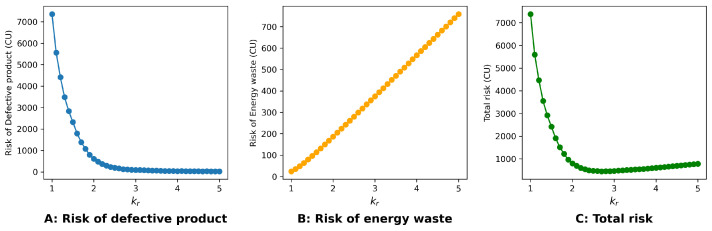
Cumulative risk in a typical week when $K_{r}\in$ [1.0–5.0].

As shown in Figure 8, the system faces the lowest cumulative risk when the control parameter is selected around $K_{r}=2.5$. With more detailed information provided in Table 2, the best control parameter is $K_{r}=2.7$.

#### 4.2.2. Sensor Selection and Maintenance Reminding

**Figure 9:**
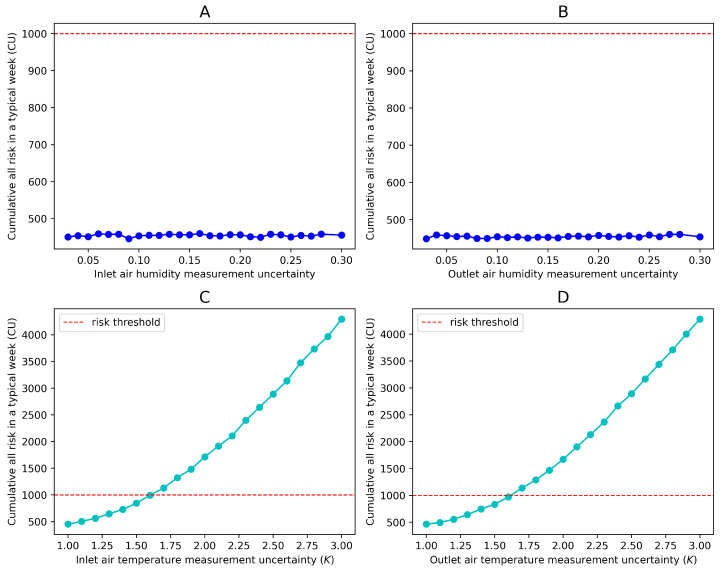
Acceptable sensor accuracy degradation under current parameters. Subfigures (**A**–**D**) respectively show the risk levels caused by different measurement uncertainties of the four sensors.

In this case study, only four sensors are considered. The budget is enough to use the best measurements. The following section will discuss the threshold of each sensor’s uncertainty in measurement. Assuming that the maximum acceptable risk of the system in a typical week is 1000 CU, and given the current risk requirements, the maximum allowable measurement uncertainty for each sensor, assuming the uncertainties of other sensors remain unchanged, is shown in Figure 9.

From the perspective of measurement uncertainty, when the latter reaches 30%, the system’s risk under typical working conditions remains significantly below 1000 CU. However, the temperature sensor’s measurement uncertainty has a greater impact on the system’s risk. Specifically, when the measurement accuracy of the air inlet and outlet temperature sensors degrades to 1.6 K, the risk threshold is reached.

#### 4.2.3. Sensor Active Fault-Tolerant Control

##### Step 1: Virtual Measurement Model

**Figure 10:**
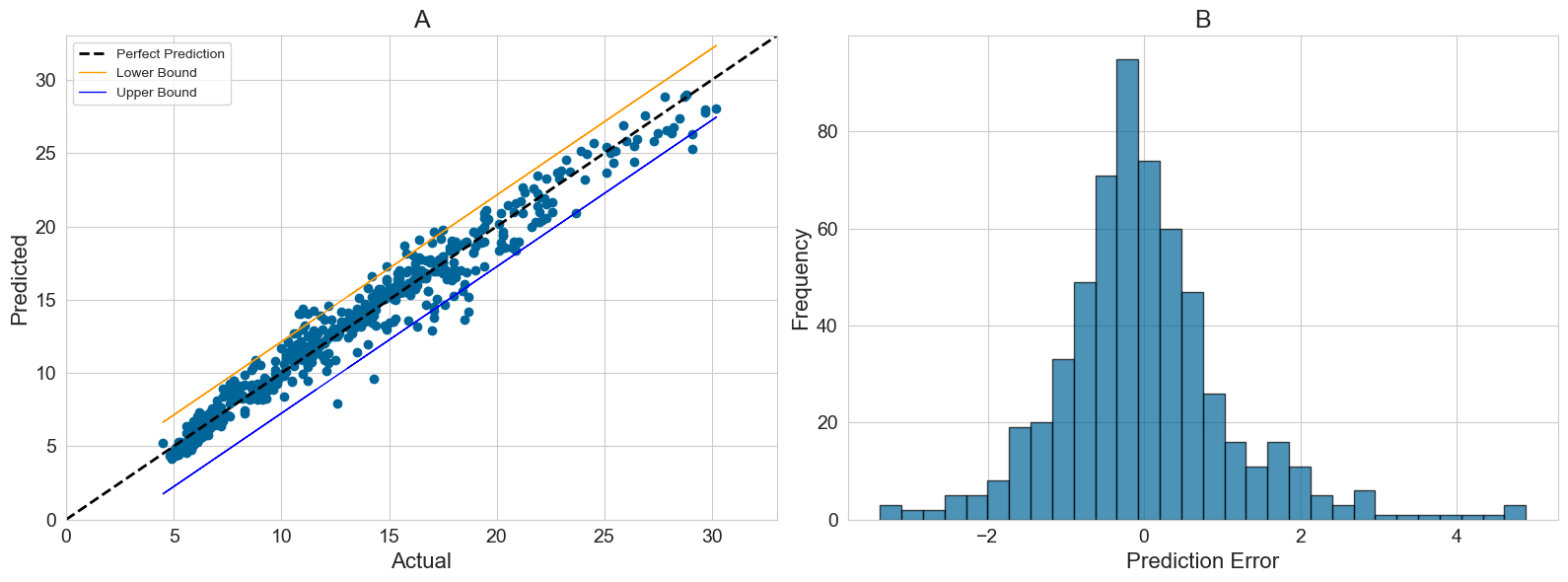
Prediction of outlet temperature.

When the outlet temperature sensor fails, predictions will be made based on typical operating condition data. Figure 10A presents the prediction results at different temperatures, where the red (+2.75 K) and blue (−2.15 K) lines represent, respectively, the 2.7% to 97.7% confidence intervals. Figure 10B shows the distribution of prediction deviations.

##### Step 2: Real-Time Fault Detection and Maximum Risk Minimization Control

As demonstrated in the sensor fault prediction in Step 1, we identified a fault in the T-out sensor at the 400th decision point.

**Figure 11:**
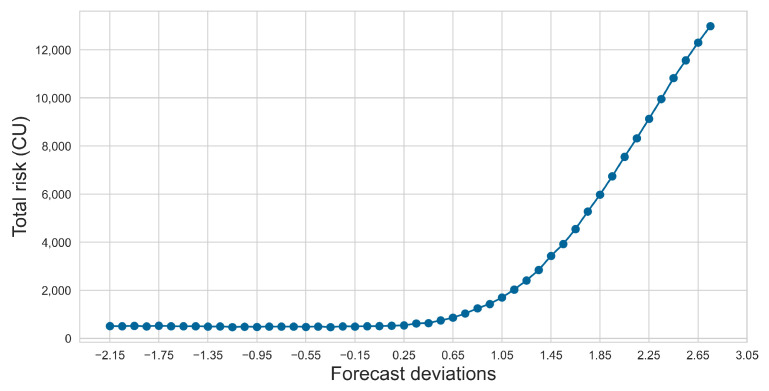
Risk with different forecast deviations.

Using the sensor imputation method from Step 1, we obtained the predicted values for the sensors, with the prediction bias lying in the interval [−2.15, 2.75]. We performed an average sampling of the prediction bias, using a step size of 0.1 K, and incorporated different measurement biases into the Monte Carlo simulation, accounting for measurement uncertainties in other parameters. This allowed us to assess the risk under various biases. As illustrated in Figure 11, the cumulative risk to the system is highest when the bias is 2.75 K.

**Figure 12:**
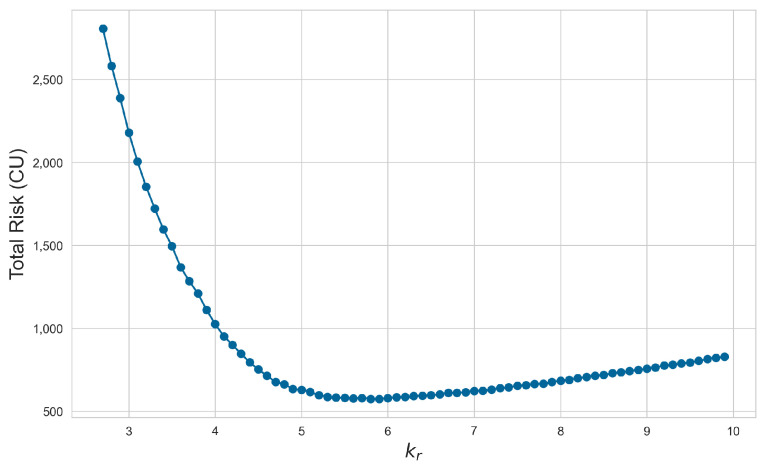
Risk evaluation with different $k_{r}$.

In the next simulation substep, we selected the sensor with the highest measurement deviation of 2.75 K and incorporated this into the risk simulation for parameter optimization. It can be concluded from Figure 12 that when $k_{r}=5.9$, the expected risk faced by the system is minimized.

### 4.3. Conclusions

This case study demonstrates how dynamic cooling system adjustment can optimize energy use in a steel plant while managing risks related to environmental conditions and sensor data quality. Through precise data collection and risk simulation, this approach effectively addresses energy wastage issues and data deviation in cooling tower ventilation adjustment. Optimized control parameters and fault-tolerance strategies ensure that system risks remain within acceptable limits under various typical operating conditions, achieving a balance between energy efficiency and risk management.

## 5. General Conclusions and Further Research

This paper presents a systematic risk assessment and alleviation framework to address decision risks caused by data quality issues in manufacturing processes. This paper provides a risk assessment framework by analyzing measurement uncertainty and sensor failures of on-site data and proposes corresponding optimization control strategies. The effectiveness and feasibility of the proposed method are demonstrated through a case study and simulation-based validation. This framework comprehensively explains how to assess the impact of data quality issues in specific decision-making scenarios and clearly outlines the necessary preparations before using the framework.

The difficulty in using this framework lies in constructing a relationship model between decision bias and risk, which typically requires a substantial amount of relevant historical data as support. When historical data are lacking, one can rely on extending existing experience or conducting experiments based on limited conditions to explore the relationship between loss and decision bias.

Potential research directions in the future include the construction of a comprehensive risk assessment model that integrates spatiotemporal dimensions, aiming to more accurately simulate and restore complex and changeable accident scenarios to improve prediction and prevention capabilities. At the same time, we are committed to the research and development of intelligent sensor technology with independent monitoring and diagnosis functions to ensure that the sensor status can be observed in real time.

## Figures and Tables

**Table 1:** Data quality characteristics of inherent and system-dependent types.

Types	Data Quality Characteristics
Inherent	Accuracy, Credibility, Completeness, Consistency, Error, Synchronicity, Variance, Objectivity, Reputation, Uncertainty
Inherent and system-dependent	Accessibility, Appropriate amount of data, Compliance, Concise representation, Ease to manipulate, Timeliness, Traceability, Understandability
System-dependent	Availability, Portability, Relevance, Interpretability, Security, Value-added

**Table 2:** Cumulative risk in a typical week when $K_{r}\in$ [2.5–3.0].

$K_{r}$	Cumulative Risk of Product Loss (CU)	Cumulative Risk of Energy Waste (CU)	Cumulative All Risk (CU)
2.5	193.90	279.51	473.41
2.6	162.03	298.78	460.80
2.7	130.76	317.70	448.47
2.8	115.69	336.90	452.58
2.9	102.03	356.02	458.05
3.0	91.45	374.96	466.41

**Figure A1:**
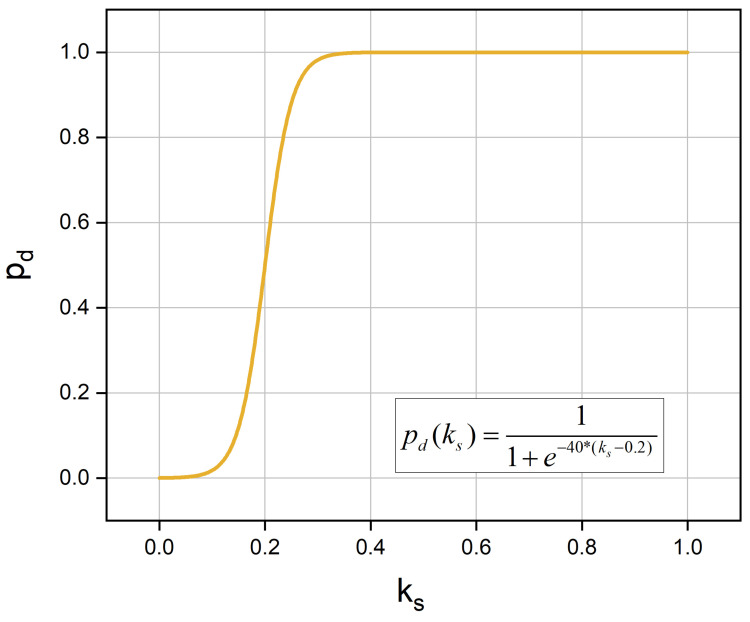
Defective probability.

## Data Availability

All data included in this study are available from the corresponding authors upon reasonable request.

## Appendix A. Control Method by Current Cooling Capacity

The water flow and air flow are constant in the cooling devices, and the worker changes the amount of the steel ingots by current calculated cooling capacity. A sensor network collects the data every 15 min, and the worker makes a decision every 15 min.

There are some typical cooling tower operating models of the open counter-flow mechanical cooling towers to determine cooling capacity, such as Merkel’s model [27], effectiveness-NTU [28], and enthalpy difference.

The predicted cooling capacity, in this case, is obtained using the enthalpy difference method. The enthalpy of moist air can be obtained by Hyland–Wexler formulations (A1)–(A3):

(A1) $$\begin{array}{cc} P_{v}= & 6.11657+7.59138\times 10^{-4}T\\ \; & +4.48027\times 10^{-7}T^{2}+1.22874\times 10^{-9}T^{3}\\ \; & +9.84205\times 10^{-13}T^{4}+4.35027\times 10^{-16}T^{5}\\ \; & -1.26197\times 10^{-19}T^{6}+1.61243\ln\left(T\right) \end{array}$$

(A2) $$d_{w}=0.6219\frac{H_{r}P_{v}}{101,326-H_{r}P_{v}}$$

(A3) $$h=1.01T+\left(2500+1.84T\right)d_{w}$$

where:

$P_{v}$ is the saturation vapor pressure of water at temperature *T*.

*T* is the temperature of the air (K).

*h* is the moist air enthalpy (kJ/kg).

$H_{r}$ is the relative humidity, $H_{t}\in \left[0,1\right]$.

$d_{w}$ is the humidity ratio (kg/kg).

1.01 is the average constant pressure specific heat kJ/(kg/K) of dry air.

1.84 is the average constant pressure specific heat kJ/(kg/K) of water vapor.

2500 is the latent heat of vaporization of water at 0 °C (kJ/kg).

According to the enthalpy difference of the humid air at the inlet and outlet, it can be known that the heat absorbed by the air per unit mass after passing through the cooling tower is (A4):

(A4) $$P=\frac{L\times (E_{\mathrm{out}}-E_{\mathrm{in}})}{v\times (1+X)}$$

where:

*P* is the heat exchange rate of the cooling tower (kJ∗kg).

*L* represents the ventilation rate of the cooling tower (kg/s).

$E_{\mathrm{in}}$ and $E_{\mathrm{out}}$ are the enthalpy values per unit mass of the air inlet and air outlet of the cooling tower, respectively (kJ/kg).

*v* is the specific volume of humid air at the measuring point ($m^{3}/\mathrm{kg}$).

*X* is the absolute humidity of the air at the measuring point ($\mathrm{kg}/m^{3}$).

According to the handbook of the cooling tower, the airflow is constant at 56.9444 $m^{3}/s$. In the standard condition (15 °C, 288.15 K; atmospheric pressure 101,325 Pa), *L* is 69.787 kg/s.

According to the history data in March 2022, the average relative humidity is 79.35%. And the average wet-bulb temperature is 12.5990 °C.

Assume that in March, the atmospheric pressure is 101,325 Pa, the average air specific volume is 0.8543 $m^{3}/\mathrm{kg}$, and the average absolute humidity is 0.0078 $\mathrm{kg}/m^{3}$.

In the standard condition (15 °C, 288.15 K; atmospheric pressure 101,325 Pa), *L* is 69.787 kg/s.

In order to reduce the defective rate, the cooling capacity can be increased through redundant cooling. The redundancy factor is expressed in Equation (A6):

The steel brings heat to the cooling system with the input power $P_{\mathrm{in}}$. Therefore, keeping the balance between the heat power $P_{\mathrm{in}}$ from the steel and the cooling power $P_{\mathrm{need}}$ is essential for the quenching pool, which is expressed in Equation (A5).

(A5) $$P_{\mathrm{need}}=P_{\mathrm{in}}$$

The measurement cooling power $P_{\mathrm{need}}$ is calculated by Equation (A4) with sensor data. There are potential quality issues in the sensor data.

The real cooling power $P_{\mathrm{real}}$ is calculated through Equation (A4) too, but with the real value of the temperature and humidity.

To reduce the defective rate, one can increase the cooling capacity through providing redundant cooling air. The redundancy coefficient is expressed in Equation (A6):

(A6) $$k_{r}=v_{\mathrm{real}}/v_{\mathrm{need}}$$

## Appendix B. Risk Identification and Calculation

Many risks exist in the considered system, caused by mechanical failure, imprecise control, or data quality issues. Two risks caused by data quality issues are considered in this case study. The first risk $r_{1}$ is the product becoming defective because of uncertain control. The steel quality is sensitive to temperature changes, which affect metallography and thus the product quality. The second risk $r_{2}$ is energy wasted for cooling.

### Appendix B.1. Risk of Defective Product

The cooling shortage is $k_{s}$:

(A7) $$k_{s}=\frac{P_{\mathrm{real}}-P_{\mathrm{in}}}{P_{\mathrm{real}}}$$

One assumes that the defective probability can be obtained by Equation (A8) with cooling shortage $k_{s}$ in each control cycle. Figure A1 shows the relation between the defective rate $P_{d}$ and the cooling shortage $k_{s}$.

(A8) $$p_{d}\left(k_{s}\right)=\frac{1}{1+e^{-40*(k_{s}-0.2)}},k_{s}\in \left[0,1\right]$$

Let us define the cost unit by CU. The loss is $c_{d}=100\;\mathrm{CU}$ when all products are defective in a control period, which is spent on reprocessing. According to Equation (5), the defective production risk in one control is expressed in Equation (A9):

(A9) $$r_{d}=p_{d}*c_{d}=\frac{100}{1+e^{-40*(k_{s}-0.2)}},k_{s}\in \left[0,1\right]$$

The total risk $Risk_{d}$ in one month can be obtained by Equation (A10):

(A10) $$Risk_{d}=\sum r_{d}$$

### Appendix B.2. Risk of Cooling Power Waste

When the real cooling power is bigger than the demand, it means that some energy used for cooling is wasted.

When $P_{\mathrm{real}}>P_{\mathrm{in}}$, the probability $p_{\mathrm{ew}}$ of energy waste is 100%. The lost $c_{\mathrm{ew}}$ (CU) is estimated in Equation (A11). Assuming the unit price of cooling power is 0.003 USD/kWh (including the electricity price and cooling tower maintenance cost, when an 11 kW fan brings 3050 kW average cooling power)

(A11) $$c_{\mathrm{ew}}=0.003*\frac{P_{\mathrm{real}}-P_{\mathrm{in}}}{3600*1000}$$

So the risk of waste cooling power $r_{\mathrm{ew}}$ can be obtained using Equation (A12):

(A12) $$r_{\mathrm{ew}}=p_{\mathrm{ew}}*c_{\mathrm{ew}}=0.003*\frac{P_{\mathrm{real}}-P_{\mathrm{in}}}{3600*1000}$$

The total risk $Risk_{\mathrm{ew}}$ in one month can be obtained using Equation (A13):

(A13) $$Risk_{\mathrm{ew}}=\sum r_{\mathrm{ew}}$$

The system risk $Risk_{\mathrm{total}}$ in this month can be obtained by Equation (A14):

(A14) $$Risk_{\mathrm{total}}=Risk_{d}+Risk_{\mathrm{ew}}=\sum r_{d}+\sum r_{\mathrm{ew}}$$
